# Disparities in testicular cancer incidence, mortality, and place of death trends from 1999 to 2020: A comprehensive cohort study

**DOI:** 10.1002/cnr2.1880

**Published:** 2023-08-16

**Authors:** Beck O. Gold, Anushka Ghosh, Saveli I. Goldberg, Fumiko Chino, Jason A. Efstathiou, Sophia C. Kamran

**Affiliations:** ^1^ Department of Radiation Oncology Massachusetts General Hospital, Harvard Medical School Boston Massachusetts USA; ^2^ Department of Radiation Oncology Memorial Sloan Kettering Cancer Center New York New York USA

**Keywords:** incidence, metastatic neoplasm, mortality, place of death, testicular cancer

## Abstract

**Background:**

Testicular cancer (TC) mortality rates have decreased over time, however it is unclear whether these improvements are consistent across all communities.

**Aims:**

The aim of this study was to analyze trends in TC incidence, mortality, and place of death (PoD) in the United States between 1999–2020 and identify disparities across race, ethnicity, and geographic location.

**Methods and Results:**

This cross‐sectional study used CDC WONDER and NAACCR, to calculate age‐adjusted rates of TC incidence and mortality, respectively. PoD data for individuals who died of TC were collected from CDC WONDER. Using Joinpoint analysis, longitudinal mortality trends were evaluated by age, race, ethnicity, US census region, and urbanization category. TC stage (localized vs metastatic) trends were also evaluated. Univariate and multivariate regression analysis identified demographic disparities for PoD. A total of 8,456 patients died of TC from 1999–2020. Average annual percent change (AAPC) of testicular cancer‐specific mortality (TCSM) remained largely stable (AAPC, 0.4; 95% CI −0.2 to 0.9; *p* = 0.215). Men ages 25–29 experienced a significant increase in TCSM (AAPC, 1.3, *p* = 0.003), consistent with increased metastatic testicular cancer‐specific incidence (TCSI) trend for this age group (AAPC, 1.6; *p* < 0.01). Mortality increased for Hispanic men (AAPC, 1.7, *p* < 0.001), with increased metastatic TCSI (AAPC, 2.5; *p* < 0.001). Finally, younger (<45), single, and Hispanic or Black men were more likely to die in medical facilities (all *p* < 0.001). The retrospective study design is a limitation.

**Conclusion:**

Significant increases in metastatic TC were found for Hispanic men and men aged 25–29 potentially driving increasing testicular cancer specific mortality in these groups. Evidence of racial and ethnic differences in place of death may also highlight treatment disparities.

## 1 INTRODUCTION

Testicular cancer (TC) is the most common malignant neoplasm among men between the ages of 15–35.^1^, ^2^, ^3^ In the general population, however, TC is a rare cancer that accounts for 0.5% of all new cancers (~9910 cases) and 0.1% of all cancer deaths (~460 deaths) in the United States (US) in 2022.^4^ While the overall prognosis from TC is excellent (5‐year survival rate above 95%),^5^ an increase in incidence in the US has been reported over the last few decades.^2^, ^6^ The factors behind rising cases are unclear; studies suggest that increased surveillance and risk factors, like cryptorchidism, are unlikely to explain this trend.^7^, ^8^ Thankfully, despite an overall increase in incidence, mortality rates among all TC patients generally remain stable.^3^, ^9^

Yet, as with many cancer disparities, which are driven by differential access and structural racisms, TC mortality and incidence rates are not uniform across race and ethnicity.^2^, ^10^ In a recent analysis of disparities and trends of genitourinary incidence and mortality in the US, it was found that, while testicular cancer‐specific incidence (TCSI) increased in each racial and ethnic group between 2000 and 2019, corresponding testicular cancer‐specific mortality (TCSM) increased for Hispanic men and decreased for White men.^9^ Additional study with a modern, comprehensive evaluation of incidence and mortality rates by age and place of death (PoD) trends among TC patients can further elucidate discrepancies between racial, ethnic, and age groups.

This study aims to identify trends and disparities in TCSM by various demographic characteristics, including age, race, and ethnicity, between 1999 and 2020 using contemporary comprehensive population‐based nationwide data. In addition, we analyzed PoD of TC patients in the context of additional socioeconomic factors, such as marital status and education level. Finally, we evaluated US trends in localized and metastatic TCSI rates to better understand the impact on TCSM rates.

## 2 METHODS

### 2.1 Data sources

The Centers for Disease Control and Prevention (CDC) Wide‐ranging Online Data for Epidemiologic Research (WONDER), maintained by the National Center for Health Statistics, contains deidentified mortality and population data of all individuals in the US, including underlying cause of death and demographic information.^11^ Demographic characteristics include age, sex, PoD, race, ethnicity, US census regions, and urbanization category. Cancer incidence evaluated by race and ethnicity in the context of localized and metastatic TC diagnoses was obtained from the North American Association of Central Cancer Registries (NAACCR) database.^12^ This population‐based data set provides comprehensive cancer incidence for North America, including stage at diagnosis. Analysis was limited to NAACCR data from the US. Due to secondary analysis of publicly available deidentified data, the Mass General Brigham institutional review board considered this study exempt from human participant research guidelines.

### 2.2 Study design

The CDC WONDER database was queried for individuals who died of TC between 1999 and 2020. Cause of death was based on the *International Statistical Classification of Diseases and Related Health Problems*, *Tenth Revision* code (testicular cancer code C62). Age‐adjusted rates of death were obtained, along with demographic characteristics as described in Data Sources. Age‐adjusted rates per 100 000 population were calculated from the crude rate (count/population × 100 000) and subsequently weighted by the proportion of the persons in the corresponding age groups to the standard population (2000 US standard population).

Age at time of death was reported in 5‐year increments starting from birth up to age 75+. CDC Wonder censors data for groups with <10 deaths per year and therefore the trend analysis was limited to ages 20–54 given low testicular mortality in younger and older groups. Urbanization categories and US Census regions are described in eTables 1 and 2 in the Supplement and are consistent with the 2013 National Center for Health Statistics. CDC WONDER defines mutually exclusive race categories as: White, Black, American Indian or Alaska Native (AIAN), and Asian or Other Pacific Islander (API). Ethnicity was defined as Hispanic or non‐Hispanic. For TCSM and TCSI trend analysis, race/ethnicity was defined as Hispanic, non‐Hispanic White (NHW), and non‐Hispanic Black (NHB). Trends among API and AIAN racial groups were not analyzed due to a paucity of data. NAACCR data were used to assess the age‐adjusted incidences of localized and metastatic TCs in the US at the time of diagnosis per 100 000 men for the years 1999–2018 (based on data availability). Localized rates were analyzed for ages 20–85+, while metastatic incidence rates were analyzed for ages 20–54. Data for other ages were not retrievable due to low incidence frequency for those age groups.

### 2.3 Statistical analysis

Analysis was performed from August 2021 to September 2022. Trends in TCSM and TCSI were assessed using Joinpoint regression to estimate average annual percent changes (AAPCs).^13^ When needed, annual percent changes (APCs) were used to identify calendar years during which the course of incidence and mortality trends demonstrates significant shifts in trend. A maximum four join points were allowed in fitting the data. TCSM trends were analyzed by race, ethnicity, 5‐year age groups, urbanization category, and US Census region. Both localized and metastatic TCSI trends were analyzed by race, ethnicity, and 5‐year age groups. Rates are reported per 100 000 population each year. Data analysis was performed using Joinpoint Regression Program, Version 4.9.1.0.

To evaluate trends in PoD among TC patients, a univariate binary logistic regression analysis used individual data files for the years 1999 to 2020. Hospice facility deaths were not documented until 2003. Independent variables, including age group, marital status, education level, race, and ethnicity, were used to define associations between these independent variables. The year of death was included as a continuous variable. Logistic regression analysis was done by SAS 9.4, and univariate and multivariate models were reported using odds ratios (OR).

All *p*‐values are based on a two‐sided hypothesis test with values less than .05 considered statistically significant.

## 3 RESULTS

Aggregate data for 8456 men who died of TC in the US from 1999 to 2020 were collected. Among these, 7643 (90.4%) were White men, 537 (6.35%) were Black men, 192 (2.27%) were API men, and 84 (1.01%) were AIAN men. There were 1553 (18.4%) deaths among Hispanic men, 6882 (81.4%) deaths among non‐Hispanic men, and 21 (0.25%) deaths for unknown ethnic identities. The largest proportion of TC deaths was observed in men between the ages 25–39 (3088 [36.5%]) (eTable 3).

### 3.1 TCSM by age

**FIGURE 1:**
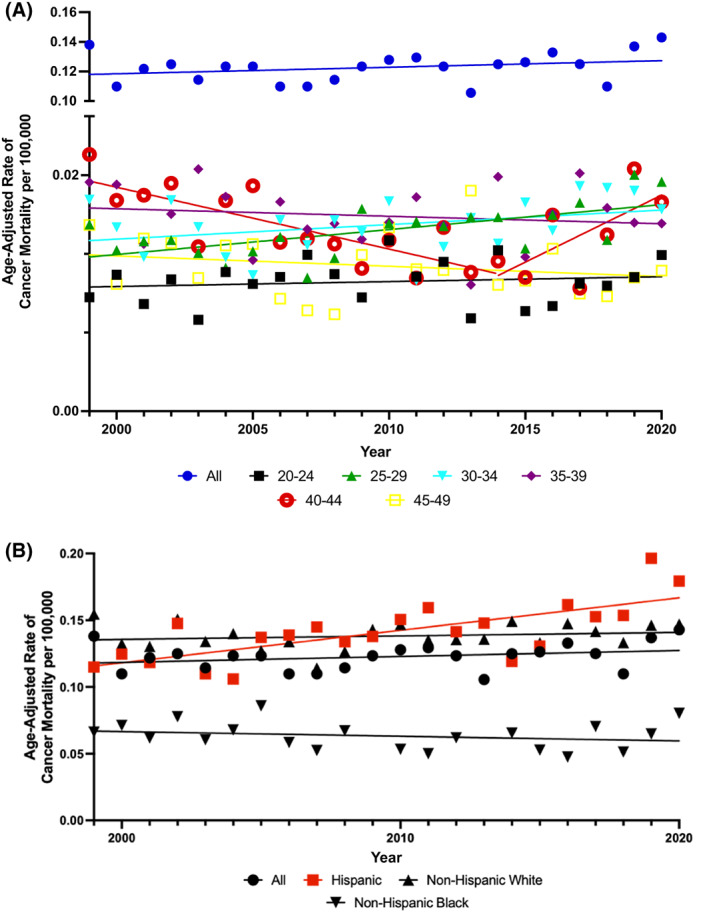
Age‐adjusted rates of testicular cancer‐specific mortality (TCSM) per 100 000 Population by (A) 5‐year age group and (B) race and ethnicity. Trends in age‐adjusted TCSM rates by (A) 5‐year age group; (B) race and ethnicity. Solid lines represent Joinpoint modeled rates and markers represent observed rates. Mortality data are from Centers for Disease Control and Prevention (CDC) Wide‐ranging Online Data for Epidemiologic Research (WONDER) database.

Among all age groups, TCSM remained steady over the 20‐year study period. Among men aged 25–29, the TCSM rate significantly increased (AAPC, 1.3; 95% CI, 0.5–2.2; *p* = .003). For the 40–44 age group prior to 2015, the TCSM rate decreased significantly (AAPC, −3.2; 95% CI, −4.1 to −1.5; *p* = .001). After 2015, the TCSM rate increased, although this trend was not significant. There were a number of age groups, (20–24, 30–34, and 50–54), that experienced a general increase in TCSM rates across the study period, although these were not statistically significant (Figure 1A and eTable 4).

### 3.2 TCSM by race and ethnicity

Upon evaluation of TCSM by race and ethnicity, Hispanic men had a statistically significant increase in overall TCSM rate (AAPC, 1.7; 95% CI, 0.9–2.5; *p* < .001). Rate of TCSM for NHW individuals increased, while NHB individuals experienced a decrease although neither of these were statistically significant (Figure 1B and eTable 4).

### 3.3 TCSM trends across census regions and urbanization categories

There were no statistically significant trends in TCSM rates among US Census regions. Over the 20‐year study period, the TCSM rate decreased in the Northeast, while it increased in the South, Midwest, and West (eFigure 1 and eTable 4).

Among urbanization categories, there were no statistically significant trends in TCSM. Large Central Metro and Noncore category TCSM rates decreased from 1999 to 2020 while all other categories, including Large Fringe, Medium Metro, Small Metro, and Micropolitan increased (eFigure 2 and eTable 4).

### 3.4 Trends in TCSM by PoD

Out of 8456 patients who died of TC, there were 6926 patients with known PoD from 1999 to 2020 (eTable 5). Among these 6926 patients, trends in PoD were evaluated for five age groups (birth‐24, 25–44, 45–64, 65–84, and 85+), marital status, education level, race, and ethnicity. Upon univariable and multivariable logistic regression analysis on this subset of patients, modeled for home or hospice versus medical facility PoD, older patients (those aged 65–84 [OR, 1.82; 95% CI, 1.51–2.18] and those aged 85+ [OR, 2.27; 95% CI, 1.54–3.36]) were at higher odds of dying at home or hospice (all *p* < .001). Married individuals had higher odds of home or hospice death (OR, 1.48; 95% CI, 1.31–1.66, *p* < .001). Specific groups were significantly less likely to have a home or hospice death, including Hispanic, (OR, 0.60; 95% CI, 0.52–0.69), AIAN (OR, 0.43; 95% CI, 0.25–0.70), or Black patients (OR, 0.57; 95% CI, 0.46–0.72) (all *p* < .005) (Table 1).

**TABLE 1:** Univariate and multivariate analysis for place of death.

Modeled response: home + hospice versus medical facility				
	Univariate		Multivariate	
	OR (95% CI)	*p*	OR (95% CI)	*p*
Age group, y				
Birth‐24	0.78 (0.67–0.91)	.0022	0.95 (0.80–1.13)	.5741
25–44 (reference)	1.00		1.00	
45–64	1.21 (1.08–1.36)	.0009	1.04 (0.92–1.18)	.4866
65–84	2.3 (1.92–2.67)	<.0001	1.82 (1.51–2.18)	<.0001
> 85	2.80 (1.97–4.00)	<.0001	2.27 (1.54–3.36)	<.0001
Year of death (continuous variable)	1.03 (1.03–1.04)	<.0001	1.04 (1.03–1.05)	<.0001
Marital status				
Single (reference)	1.00		1.00	
Married	1.70 (1.53–1.89)	<.0001	1.48 (1.31–1.66)	<.0001
Widowed	1.93 (1.42–2.63)	<.0001	1.11 (0.77–1.58)	.5839
Divorced/separated	1.24 (1.06–1.45)	.0081	1.09 (0.92–1.30)	.3236
Educational level				
Some high school or less (reference)	1.00		1.00	
High School graduate (>4y)	1.16 (1.02–1.32)	.0253	1.05 (0.92–1.20)	.4609
Some College/Associate's degree	1.30 (1.11–1.51)	.0008	1.12 (0.96–1.31)	.1517
College graduate (>4 y)	1.23 (1.04–1.45)	.0149	1.00 (0.84–1.19)	.9782
Advanced degree	1.36 (0.99–1.87)	.0578	0.82 (0.59–1.15)	.2513
Race				
White (reference)	1.00		1.00	
Black or African American	0.61 (0.49–0.75)	<.0001	0.57 (0.46–0.72)	<.0001
Asian or Pacific Islander	0.71 (0.51–0.99)	.0409	0.72 (0.51–1.00)	.0521
American Indian or Alaska Native	0.49 (0.29–0.82)	.007	0.43 (0.25–0.74)	.0023
Ethnicity				
Hispanic or Latino	0.60 (0.53–0.69)	<.0001	0.60 (0.52–0.69)	<.0001
Non‐Hispanic or Latino (reference)	1.00		1.00	

### 3.5 Metastatic and localized TCSI

**FIGURE 2:**
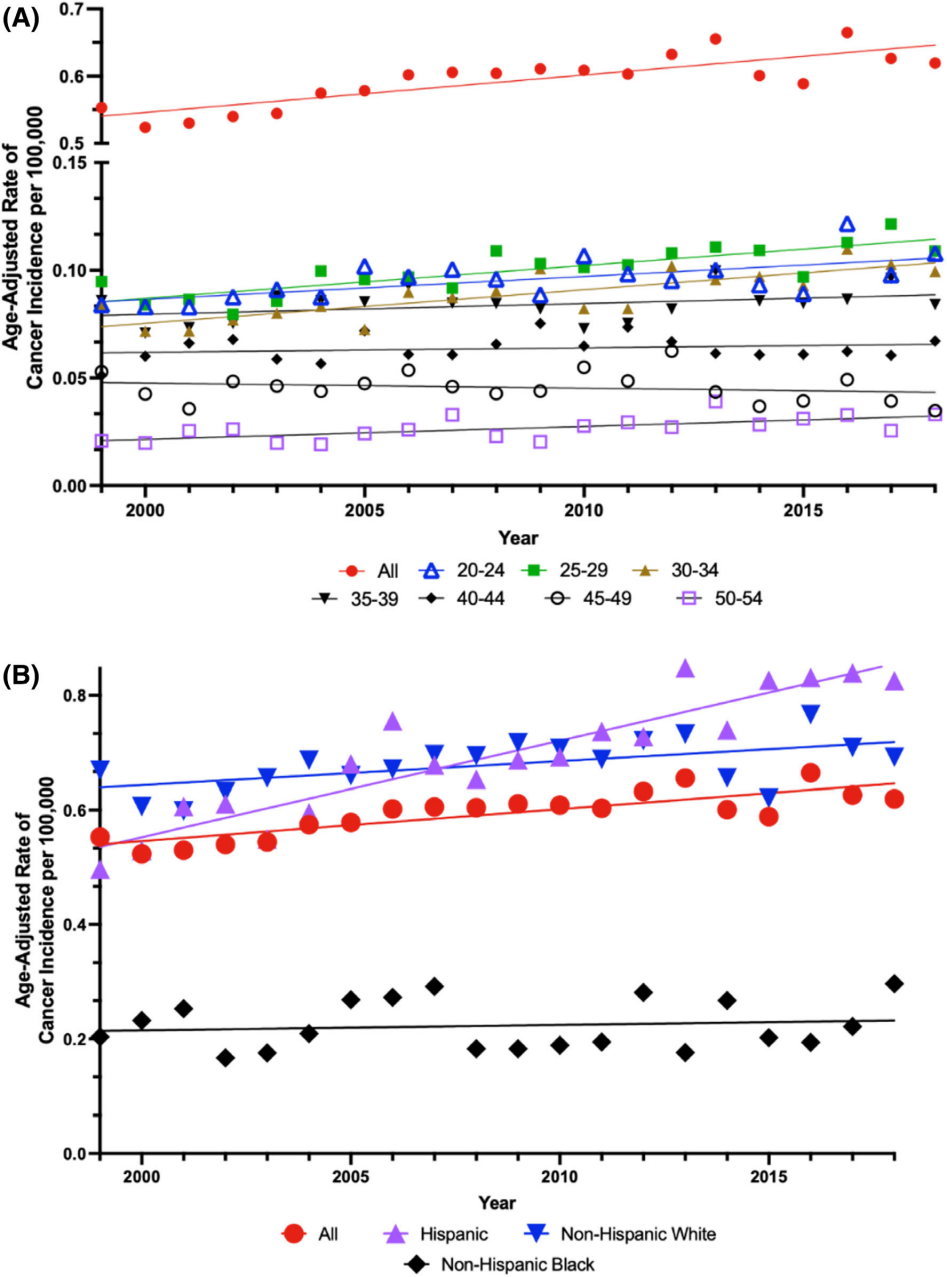
Age‐adjusted rates of metastatic testicular cancer‐specific incidence per 100 000 population by (A) 5‐year age groups and (B) race and ethnicity. Trends in metastatic TCSI by (A) 5‐year age groups and (B) race and ethnicity. Solid lines represent Joinpoint modeled rates and markers represent observed rates. Incidence data are from the North American Association of Central Cancer Registries (NAACCR) database.

Metastatic TCSI rates for all ages increased consistently throughout the study period (AAPC, 1.0; 95% CI, 0.4–1.6; *p* < .001) (Figure 2A and eTable 6). Upon breakdown by age group, patients aged 20–24 (AAPC 1.1; 95% CI 0.5–1.7), 25–29 (AAPC, 1.6; 95% CI 1.0–2.1), 30–34 (AAPC, 1.8; 95% CI 1.2–2.4), and 50–54 (AAPC, 2.3; 95% CI 1.0–3.6) had a statistically significant increase in metastatic TCSI rates (all *p* ≤ .001). The only metastatic TCSI rate that decreased during the study period was the 45–49 age group, although this trend was not statistically significant. Upon evaluation of metastatic TCSI rates across racial and ethnic groups, Hispanic men had the largest rate increase (AAPC, 2.5; 95% CI, 2.0–3.1; *p* < .001), followed by NHW men (AAPC, 0.6; 95% CI, 0.2–1.1, *p* = .009), while NHB men demonstrated no statistically significant increase in metastatic TCSI rates (Figure 2B).

Localized TCSI was analyzed to provide a comprehensive understanding of the current trends in TC. From 1999 to 2018, there was a slight decrease in localized TCSI rate across the entire study period that was not statistically significant. Age‐adjusted localized TCSI rates by age, race, and ethnicity can be found in eTable 7.

## 4 DISCUSSION

This contemporary, comprehensive analysis of TCSM trends provides an understanding of disparities among men dying of TC in the United States. Although TC mortality rates are decreasing overall, we found significant racial, ethnic, and age group differences in mortality, metastatic incidence, and PoD over the study period, specifically among men aged 25–29 and Hispanic men. These results may be useful for institutions, clinicians, and epidemiologists, to improve TC care and implement targeted policies for TC screening, treatment and education in specific communities.

Prior studies investigating TC mortality across various demographic variables support our findings. TC mortality rates, in general, have been found to be highest among the 20–44 age group.^1^, ^3^, ^5^, ^7^, ^14^, ^15^, ^16^, ^17^ However, in stratifying by 5‐year age groups, our study found that, in 2020, compared to 2000, 30 more men aged 25–29 died of TC. It is unclear why this age group experienced the highest increase compared to other age groups, but prior data have found that younger patients are more likely to experience financial hardships post‐cancer diagnosis,^18^ and this alone may contribute to less frequent visits to the doctor or adherence to treatment plans,^19^ thus increasing mortality. Separately, a 2011 study projected that national cancer costs were to increase by 27%, and a portion of the increased cost falls on the responsibility of the patient.^20^ Extreme financial distress can contribute to mortality through poorer well‐being, impaired health‐related quality of life, and sub‐par quality of care.^21^ Additional contributing factors to the increased TCSM rates in this young age group include lower likelihood of routine wellness visits for men in this age group, and the general stigma regarding testicular self‐examinations and symptoms.^22^, ^23^

We observed that Hispanic men experienced an increase in TCSM rates across the entire 20‐year study period. A recent analysis by Schaefer et al. also noted increasing TCSM rates among the Hispanic population between 1990 and 2020.^9^ Potential explanations include the observation that Hispanic individuals are generally more likely than NHWs to be diagnosed at later cancer stages.^24^, ^25^, ^26^ Prior studies have explored the association between late‐stage diagnoses and lower socioeconomic status among Hispanic patients,^27^ which can overall contribute to increased deaths. Our findings of increasing TCSM rates among men aged 25–29 and among Hispanic men are consistent with our observation of increased rates of metastatic incidence among these same groups. It is known that early detection of cancer is paramount for favorable cancer outcomes.^28^, ^29^

Our findings may additionally be related to lack of knowledge and stigma surrounding TC in these specific demographic cohorts. A study by Casson and Roy of men aged 18–45 found that only 39% of respondents knew that TC was the most common malignancy for their age group, and a striking minority (17%) had previously heard of testicular self‐examination.^30^ This lack of knowledge and awareness could potentially explain the increasing metastatic diagnoses for men between 20 and 34 in our study as well as the corresponding increase in TC death among men between 25 and 29. Further, studies have shown that men from underrrepresented communities are much less likely to get appropriate preventative screenings, and equity in patient education remains a challenge in patient‐provider relationships.^31^, ^32^

PoD is an essential component of end‐of‐life care and one that is seldom mentioned in the context of mortality‐based analyses.^33^ Research has shown that patients prefer to die in the location of their choice, which is most often at home.^34^, ^35^, ^36^ In a study by Bell et al,^37^ congruence between an individual's preferred PoD and their actual PoD may be as low as 30%, meaning the minority of patients have goal concordant care at the end of life. Lower congruence was associated with patients who were single, lived alone, and racial and ethnic minorities.^37^, ^38^ Our results are consistent with prior analyses in that patients <65 years of age and those who were single had higher odds of medical facility death.^39^, ^40^ Particularly concerning are our findings that Hispanic men also have increased odds of dying in a medical facility, as this is compounded by the increasing TC mortality rates among this population seen over the last 20 years.

### 4.1 Limitations

Study limitations are primarily due to the CDC WONDER and NAACCR population‐based datasets. Important cancer related variables, such as disease stage at diagnosis, metastatic locations, histopronostic group, cancer treatment course, and time from diagnosis to death are not captured. Additionally, as patient deaths must be reported to the CDC, missing data or other inaccuracies could be present. Such inaccuracies could include misidentified cause of death, as it relies on the discretion of the health care provider. Thus, like other epidemiologic studies, it is difficult to draw definitive conclusions about the cause of observed trends. Future studies should further explore the role of socioeconomic status, lack of education/awareness, and uninsurance on TCSM rates. Regarding PoD data, the study tracked location of death rather than facility enrollment. Hence, it is possible that patients being treated at home, or a hospice facility, may have transferred to a hospital in their final hours. Additionally, PoD was categorized broadly by location, which may not capture all end‐of‐life care services at the facility. For instance, patients who received palliative or hospice care before death at a hospital will still be categorized as a medical facility death. Another limitation includes limited TC mortality data for patients over the age of 55. However, TC affects mainly adolescent and young adult patients, and lower mortality frequency for older populations is not surprising. Finally, the term, “men,” is used throughout the paper for ease of understanding, but we recognize that gender is a spectrum, and individuals of many gender identities are implicitly included in this discussion of TC.

## 5 CONCLUSION

In summary, our analysis of a comprehensive national database demonstrated that significant racial and ethnic disparities exist with respect to TC mortality, incidence, and PoD. Specifically, this study highlights the need to explore the variables associated with increasing TCSM and metastatic TCSI rates among Hispanic men, as well as men aged 25–29. Further, our analyses indicate the need to continue advocating for patients in their final stages of disease so that younger patients, as well as racial and ethnic minorities, pass in their preferred place. Longer follow‐up is needed to determine whether these concerning trends persist in these demographic cohorts. Finally, this highlights the need for targeted education and policies to prevent worsening TC inequities among these vulnerable communities.

## AUTHOR CONTRIBUTIONS

**Beck Gold:** Conceptualization (equal); data curation (equal); formal analysis (equal); investigation (equal); methodology (equal); visualization (equal); writing – original draft (equal); writing – review and editing (equal). **Anushka Ghosh:** Conceptualization (equal); data curation (equal); formal analysis (equal); investigation (equal); writing – review and editing (equal). **Saveli Goldberg:** Formal analysis (equal); investigation (equal); visualization (equal); writing – review and editing (equal). **Fumiko Chino:** Methodology (equal); project administration (equal); supervision (equal); writing – review and editing (equal). **Jason A Efstathiou:** Project administration (equal); supervision (equal); writing – review and editing (equal). **Sophia C. Kamran:** Conceptualization (equal); data curation (equal); formal analysis (equal); investigation (equal); methodology (equal); project administration (lead); resources (equal); supervision (equal); visualization (equal); writing – original draft (equal); writing – review and editing (equal).

## FUNDING INFORMATION

None.

## CONFLICT OF INTEREST STATEMENT

The authors have stated explicitly that there are no conflicts of interest in connection with this article.

## ETHICS STATEMENT

Due to secondary analysis of publicly available deidentified data, the institutional review board considered this study exempt from human participant research guidelines.

## Supporting information


**Data S1:** Supporting Information.

## Data Availability

The data underlying this article are available via the publicly available Centers for Disease Control and Prevention Wide‐ranging Online Data for Epidemiologic Research (CDC‐WONDER) found here: https://wonder.cdc.gov/.
