# Supplementary material for: Disparities in testicular cancer incidence, mortality, and place of death trends from 1999 to 2020: A comprehensive cohort study
Source: Cancer Rep (Hoboken). 2023 Aug 16;6(10):e1880. doi: 10.1002/cnr2.1880 (PMC10598251; doi:10.1002/cnr2.1880)
Supplement: Supplementary file 1 — Data S1: Supporting Information. [file CNR2-6-e1880-s001.docx]

**Supplementary Online Content**

eTable 1. 2013 US County and County Equivalent Urbanization

eTable 2. Census Region and Associated States

eTable 3. Testicular Cancer Mortality in the US by Age, Race, Ethnicity, Marital Status, Education Level, and Place of Death

eTable 4. Annual Percentage Changes in Testicular Cancer Death Rates by Age, Race, Ethnicity, Census Region, and Urbanization, 1999-2020

eTable 5. Testicular Cancer Mortality Place of Death in the US by Age, Marital Status, Educational Level, Race, and Ethnicity 1999-2020

eTable 6. Annual Percentage Changes in Metastatic Testicular Cancer Incidence Rates by Age, Race, and Ethnicity, 1999-2018

eTable 7. Annual Percentage Changes in Localize

d Testicular Cancer Incidence Rates by Age, Race, and Ethnicity, 1999-2018

eFigure 1. Age-Adjusted Rate of Testicular Cancer–Specific Mortality (TCSM) Over Time by Census Region

eFigure 2. Age-Adjusted Rate of Testicular Cancer-Specific Mortality (TCSM) Over Time by Urbanization Area

**eTable 1.** 2013 US County and County Equivalent Urbanization: Classification as Metropolitan or Non-metropolitan and Subclassified into Six Urbanization Levels Used for Analysis. Urbanization levels are determined by their metropolitan statistical area (MSA) derived from 2012 post-census estimates

| **County Classification** | | | | | | |
| --- | --- | --- | --- | --- | --- | --- |
|  | **Metropolitan** | | | | **Non-Metropolitan** | |
| **Urbanization Level** | Large Central Metro | Large Fringe Metro | Medium | Small | Micropolitan | Non-Core |
| **MSA Population** | ≥1,000,000 | 1,000,000 | 250,000 – 999,999 | <250,000 | 10,000 – 49,000 | * |
| **Number of Counties** | 68 | 368 | 373 | 358 | 641 | 1335 |
| **Distribution of U.S Resident Population (%)** | 30.5 | 24.7 | 20.9 | 9.2 | 8.7 | 6.1 |

**eTable 2.** Census Region and Associated State: 50 States and Washington D.C. Are Classified Into four Regions Across the United States

| **Census Region** | | | |
| --- | --- | --- | --- |
| Northeast | Midwest | South | West |
| Connecticut | Illinois | Alabama | Alaska |
| Maine | Indiana | Arkansas | Arizona |
| Massachusetts | Iowa | Delaware | California |
| New Hampshire | Kansas | Distict of Columbia | Colorado |
| New Jersey | Michigan | Florida | Hawaii |
| New York | Minnesota | Georgia | Idaho |
| Pennsylvania | Missouri | Kentucky | Montana |
| Rhode Island | Nebraska | Louisiana | Nevada |
| Vermont | North Dakota | Maryland | New Mexico |
|  | Ohio | Mississippi | Oregon |
|  | South Dakota | North Carolina | Utah |
|  | Wisconsin | Oklahoma | Washington |
|  |  | South Carolina | Wyoming |
|  |  | Tennessee  Texas  Virginia  West Virginia |  |

| eTable 3. Testicular Cancer Mortality in the US by Age, Race, Ethnicity, Marital Status, Education Level, and Place of Death | | | | | | | | | | | | |
| --- | --- | --- | --- | --- | --- | --- | --- | --- | --- | --- | --- | --- |
| **Year** | 1999 | 2000 | 2001 | 2002 | 2003 | 2004 | 2005 | 2006 | 2007 | 2008 | 2009 | 2010 |
| **Total n (%)** | 381 (4.51) | 338  (4.00) | 337 (3.99) | 394 (4.66) | 345 (4.08) | 357 (4.22) | 359 (4.25) | 364 (4.30) | 329 (3.89) | 360 (4.26) | 376 (4.45) | 399 (4.72) |
| **Age** |  |  |  |  |  |  |  |  |  |  |  |  |
| 0-19 | 7 (1.84) | 10  (2.96) | 9 (2.67) | 9 (2.84) | 10 (2.90) | 12 (3.36) | 13 (3.62) | 9  (2.47) | 7  (2.13) | 9  (2.50) | 6  (1.60) | 4  (1.00) |
| 20-24 | 27 (7.09) | 33  (9.76) | 27 (8.01) | 34 (8.63) | 25 (7.25) | 37 (10.4) | 34 (9.47) | 36 (9.89) | 43 (13.1) | 37 (10.3) | 31 (8.24) | 47  (11.8) |
| 25-29 | 51 (13.4) | 41  (12.1) | 42 (12.5) | 42 (10.66) | 39 (11.3) | 36 (10.1) | 41 (11.4) | 47 (12.9) | 36 (10.9) | 42 (11.7) | 56 (14.9) | 52  (13.0) |
| 30-34 | 52 (13.6) | 45  (13.3) | 38 (11.3) | 52 (13.2) | 46 (13.3) | 37 (10.4) | 33 (9.19) | 46 (12.6) | 38 (11.6) | 44 (12.2) | 42 (11.2) | 50  (12.5) |
| 35-39 | 55 (14.4) | 54  (16.0) | 39 (11.6) | 45 (11.4) | 54 (15.7) | 47 (13.2) | 33 (9.19) | 47 (10.7) | 41 (12.5) | 42 (11.7) | 37 (9.84) | 40  (10.0) |
| 40-44 | 59 (15.5) | 49  (14.5) | 52 (15.4) | 54 (13.7) | 39 (11.3) | 50 (14.0) | 53 (14.8) | 39 (8.24) | 40 (12.2) | 37 (10.3) | 31 (8.24) | 37  (9.27) |
| 45-49 | 43 (11.3) | 30  (8.88) | 42 (12.5) | 42 (10.7) | 34 (9.86) | 43 (12.0) | 43 (12.0) | 30 (8.24) | 27 (8.21) | 26 (7.22) | 42 (11.2) | 49  (12.3) |
| 50-54 | 19 (4.99) | 20  (5.92) | 23 (6.82) | 26 (6.60) | 27 (7.83) | 29 (8.12) | 32 (8.91) | 28 (7.69) | 23 (6.99) | 25 (6.94) | 37 (9.84) | 38  (9.52) |
| 55-59 | 16 (4.20) | 9  (2.66) | 14 (4.15) | 18 (4.57) | 12 (3.48) | 14 (3.92) | 24 (6.69) | 26 (7.14) | 20 (6.08) | 25 (6.94) | 29 (7.71) | 27  (6.77) |
| 60-64 | 11 (2.89) | 12  (3.55 | 8 (2.37) | 18 (4.57) | 10 (2.90) | 13 (3.64) | 10 (2.79) | 17 (4.67) | 14 (4.26) | 19 (5.28) | 15 (3.99) | 12  (3.01) |
| 65-69 | 11 (2.89) | 7  (2.07) | 10 (2.97) | 14 (3.55) | 10 (2.90) | 7 (1.96) | 12 (3.34) | 11 (3.02) | 9 (2.94) | 10 (2.78) | 14 (3.72) | 9  (2.26) |
| 70-74 | 10 (2.62) | 8  (2.37) | 13 (3.86) | 12 (3.04) | 9 (2.61) | 7 (1.96) | 5 (1.39) | 8 (2.20) | 8 (2.43) | 12 (3.33) | 5 (1.33) | 5  (1.25) |
| 75+ | 20 (5.25) | 20  (5.92) | 20 (5.93) | 28 (7.11) | 30 (8.70) | 25 (7.00) | 26 (7.24) | 20 (5.49) | 23 (6.99) | 32 (8.89) | 31 (8.24) | 29  (7.27) |
| All | 381 (100) | 338  (100) | 337 (100) | 394 (100) | 345 (100) | 357 (100) | 359 (100) | 364 (100) | 329 (100) | 360 (100) | 376 (100) | 399  (100) |
| **Race** |  |  |  |  |  |  |  |  |  |  |  |  |
| White | 349 (91.6) | 306  (90.5) | 304 (90.2) | 358 (90.9) | 313 (90.7) | 321 (89.9) | 313 (87.2) | 332 (91.2) | 298 (90.6) | 316 (87.8) | 354 (94.1) | 370  (92.7) |
| Black | 23 (6.04) | 25  (7.40) | 22 (6.51) | 28 (7.11) | 25 (7.25) | 25 (7.00) | 32 (8.91) | 23 (6.32) | 20 (6.08) | 26 (7.22) | 13 (3.46) | 22  (5.51) |
| API | 7  (1.84) | 3  (0.89) | 10 (2.97) | 6 (1.52) | 3 (0.87) | 9 (2.52) | 11 (3.06) | 9 (2.47) | 7 (2.13) | 15 (4.17) | 4 (1.06) | 6  (1.50) |
| AIAN | 2  (0.52) | 4  (1.18) | 1 (0.30) | 2 (0.51) | 4 (0.87) | 2 (0.56) | 3 (0.84) | 0 (0.00) | 4 (1.22) | 3 (0.83) | 5 (1.33) | 1  (0.25) |
| All | 381 (100) | 338  (100) | 337 (100) | 394 (100) | 345 (100) | 357 (100) | 359 (100) | 364 (100) | 329 (100) | 360 (100) | 376 (100) | 399  (100) |
| **Ethnicity** |  |  |  |  |  |  |  |  |  |  |  |  |
| Hispanic | 36 (9.45) | 45  (13.3) | 46 (13.6) | 58 (14.7) | 45 (13.0) | 44 (12.3) | 60 (16.7) | 64 (17.6) | 71 (21.6) | 61 (16.9) | 65 (17.3) | 70  (17.5) |
| Non-Hispanic | 338 (88.7) | 293  (86.7) | 291 (86.4) | 334 (84.8) | 296 (85.8) | 313 (87.7) | 298 (83.0) | 300 (82.4) | 258 (78.4) | 299 (83.1) | 311 (82.7) | 328  (82.2) |
| Unknown | 7 (1.84) | 0  (0.00) | 0 (0.00) | 2 (0.51) | 4 (1.16) | 0 (0.00) | 1 (0.23) | 0 (0.00) | 0 (0.00) | 0 (0.00) | 0 (0.00) | 1  (0.25) |
| All | 381 (100) | 338  (100) | 337 (100) | 394 (100) | 345 (100) | 357 (100) | 359 (100) | 364 (100) | 329 (100) | 360 (100) | 376 (100) | 399  (100) |
| eTable 3. Cont’d |  |  |  |  |  |  |  |  |  |  |  |  |
| **Year** | 2011 | 2012 | 2013 | 2014 | 2015 | 2016 | 2017 | 2018 | 2019 | 2020 | All |  |
| **Total n (%)** | 381 (4.51) | 388  (4.59) | 383 (4.52) | 411 (4.86) | 375 (4.43) | 431 (5.10) | 425 (5.03) | 401 (4.74) | 458 (5.42) | 464 (5.49) | 8456 (100) |  |
| **Age** |  |  |  |  |  |  |  |  |  |  |  |  |
| 0-19 | 7  (1.84) | 12  (3.10) | 6 (1.57) | 8 (1.95) | 5 (1.33) | 9 (2.09) | 9 (2.12) | 8 (2.00) | 8 (1.75) | 8 (1.72) | 185 (2.19) |  |
| 20-24 | 38 (9.97) | 43  (11.1) | 27 (7.05) | 47 (11.4) | 28 (7.47) | 30 (6.96) | 36 (8.47) | 35 (8.73) | 38 (8.30) | 43 (9.27) | 776 (9.18) |  |
| 25-29 | 54 (14.2) | 53  (13.7) | 55 (14.4) | 56 (13.6) | 49 (13.1) | 59 (13.7) | 64 (15.1) | 53 (13.2) | 73 (15.9) | 71 (15.3) | 1112 (13.2) |  |
| 30-34 | 32 (8.40) | 41  (10.6) | 49 (12.8) | 43 (10.5) | 54 (14.4) | 47 (10.9) | 59 (13.9) | 59 (14.7) | 59 (12.8) | 57 (12.3) | 1023 (12.1) |  |
| 35-39 | 44 (11.6) | 38  (9.79) | 26 (6.79) | 49 (11.9) | 33 (8.80) | 43 (9.98) | 53 (12.5) | 46 (11.5) | 44 (9.61) | 43 (9.27) | 953 (11.3) |  |
| 40-44 | 29 (7.61) | 40  (10.3) | 30 (7.83) | 32 (7.79) | 28 (7.47) | 40 (9.28) | 25 (5.88) | 36 (8.98) | 50 (10.9) | 44 (9.48) | 894 (10.6) |  |
| 45-49 | 37 (9.71) | 37  (9.54) | 55 (14.4) | 31 (7.54) | 32 (8.53) | 40 (9.28) | 29 (6.82) | 28 (6.98) | 32 (6.98) | 33 (7.11) | 805 (9.52) |  |
| 50-54 | 39 (10.2) | 31  (7.99) | 35 (9.14) | 38 (9.25) | 37 (9.87) | 36 (8.35) | 28 (6.59) | 22 (5.49) | 35 (7.64) | 34 (7.33) | 662 (7.83) |  |
| 55-59 | 28 (7.34) | 25  (6.44) | 29 (7.57) | 19 (4.62) | 33 (8.80) | 29 (6.73) | 29 (6.82) | 20 (4.99) | 34 (7.42) | 23 (4.96) | 503 (5.95) |  |
| 60-64 | 17 (4.46) | 17  (4.38) | 18 (4.70) | 19 (4.62) | 21 (5.60) | 20 (4.64) | 32 (7.53) | 27 (6.73) | 16 (3.49) | 22 (4.74) | 368 (4.35) |  |
| 65-69 | 9 (2.36) | 19  (4.90) | 17 (4.44) | 20 (4.87) | 16 (4.27) | 30 (6.96) | 17 (4.00) | 20 (4.99) | 22 (4.80) | 23 (4.96) | 317 (3.75) |  |
| 70-74 | 8 (2.10) | 6  (1.55) | 11 (2.87) | 16 (3.89) | 6 (1.60) | 13 (3.02) | 12 (2.82) | 22 (5.49) | 18 (3.93) | 17 (3.66) | 231 (2.73) |  |
| 75+ | 39 (10.2) | 26  (6.70) | 25 (6.53) | 33 (8.03) | 33 (8.80) | 35 (8.12) | 32 (7.53) | 25 (6.23) | 29 (6.33) | 46 (9.91) | 627 (7.41) |  |
| All | 381 (100) | 388  (100) | 383 (100) | 411 (100) | 375 (100) | 431 (100) | 425 (100) | 401 (100) | 458 (100) | 464 (100) | 8456 (100) |  |
| **Race** |  |  |  |  |  |  |  |  |  |  |  |  |
| White | 356 (93.4) | 349  (89.9) | 354 (92.4) | 365 (88.8) | 341 (90.9) | 389 (90.3) | 378 (88.9) | 361 (90.0) | 412 (90.0) | 404 (87.1) | 7643 (90.4) |  |
| Black | 20 (5.25) | 26  (6.70) | 15 (3.92) | 28 (6.81) | 23 (6.13) | 21 (4.87) | 31 (7.29) | 23 (5.74) | 29 (6.33) | 37 (7.97) | 537 (6.35) |  |
| API | 4 (1.05) | 8  (2.06) | 10 (2.61) | 9 (2.19) | 6 (1.60) | 15 (3.48) | 8 (1.88) | 11 (2.74) | 11 (2.40) | 20 (4.31) | 192 (2.27) |  |
| AIAN | 1 (0.26) | 5  (1.29) | 4 (1.04) | 9 (2.19) | 5 (1.33) | 6 (1.39) | 8 (1.88) | 6 (1.50) | 6 (1.31) | 3 (0.65) | 84 (0.99) |  |
| All | 381 (100) | 388  (100) | 383 (100) | 411 (100) | 375 (100) | 431 (100) | 425 (100) | 401 (100) | 458 (100) | 464 (100) | 8456 (100) |  |
| **Ethnicity** |  |  |  |  |  |  |  |  |  |  |  |  |
| Hispanic | 84 (0.22) | 74  (1.91) | 82 (21.4) | 65 (15.8) | 71 (84.2) | 94 (21.8) | 92 (21.6) | 97 (24.2) | 119 (26.0) | 110 (0.24) | 1553 (18.4) |  |
| Non-Hispanic | 297 (78.0) | 312  (80.4) | 300 (78.3) | 346 (84.2) | 304 (18.9) | 336 (78.0) | 332 (78.1) | 304 (75.8) | 338 (73.8) | 354 (76.3) | 6882 (81.4) |  |
| Unknown | 0 (0.00) | 2  (0.52) | 1 (0.26) | 0 (0.00) | 0 (0.00) | 1 (0.23) | 1 (0.24) | 0 (0.00) | 1 (0.22) | 0 (0.00) | 21 (0.25) |  |
| All | 381 (100) | 388  (100) | 383 (100) | 411 (100) | 375 (100) | 431 (100) | 425 (100) | 401 (100) | 458 (100) | 464 (100) | 8456 (100) |  |

|  | **eTable 4.** Annual Percentage Changes in Testicular Cancer Death Rates by Age, Race, Ethnicity, Census Region, and Urbanization from 1999 to 2020 | | | | | | |  |
| --- | --- | --- | --- | --- | --- | --- | --- | --- |
| Demographic | | Average APC from 1999-2020 |  | Segment 1 |  | Segment 2 |  |  |
| **All** | | 0.4 (-0.2, 0.4) | 0.4 (-0.2, 0.4) | 1999-2020 | NA | NA |  | |
| **Age** | |  |  |  |  |  |  |  |
| 20-24 | | 0.4 (-0.9, 1.6) | 0.4 (-0.9, 1.6) | 1999-2020 | NA | NA |  |  |
| 25-29 | | 1.3 (0.5, 2.2)** | 1.3 (0.5, 2.2)** | 1999-2020 | NA | NA |  |  |
| 30-34 | | 0.8 (-0.3, 1.8) | 0.8 (-0.3, 1.8) | 1999-2020 | NA | NA |  |  |
| 35-39 | | -0.4 (-1.5, 0.8) | -0.4 (-1.5, 0.8) | 1999-2020 | NA | NA |  |  |
| 40-44 | | -0.2 (-2.7, 2.4) | -3.2 (-4.9, -1.5)** | 1999-2015 | 10.1 (-0.6,22.0) | 2015- 2020 |  |  |
| 45-49 | | -0.7 (-2.1, 0.8) | -0.7 (-2.1, 0.8) | 1999-2020 | NA | NA |  |  |
| 50-54 | | 0.9 (-0.2, 2.1) | 0.9 (-0.2, 2.1) | 1999-2020 | NA | NA |  |  |
| **Race/Ethnicity** | |  |  |  |  |  |  |  |
| Hispanic | | 1.7 (0.9, 2.5)** | 1.7 (0.9, 2.5)** | 1999-2020 | NA | NA |  |  |
| Non-Hispanic White | | 0.2 (-0.3, 0.7) | 0.2 (-0.3, 0.7) | 1999-2020 | NA | NA |  |  |
| Non-Hispanic Black | | -0.6 (-1.8, 0.6) | -0.6 (-1.8, 0.6) | 1999-2020 | NA | NA |  |  |
| **Census Region** | |  |  |  |  |  |  |  |
| Northeast | | -0.6 (-1.7, 0.6) | -0.6 (-1.7, 0.6) | 1999-2020 | NA | NA |  |  |
| Midwest | | 0.6 (-0.0, 1.3) | 0.6 (-0.0, 1.3) | 1999-2020 | NA | NA |  |  |
| South | | 0.2 (-0.7, 1.1) | 0.2 (-0.7, 1.1) | 1999-2020 | NA | NA |  |  |
| West | | 0.5 (-0.4, 1.5) | 0.5 (-0.4, 1.5) | 1999-2020 | NA | NA |  |  |
| **Urbanization Area** | |  |  |  |  |  |  |  |
| Large Central   Metro | | -0.3 (-1.0, 0.5) | -0.3 (-1.0, 0.5) | 1999-2020 | NA | NA |  |  |
| Large Fringe Metro | | 0.3 (-0.9, 1.5) | 0.3 (-0.9, 1.5) | 1999-2020 | NA | NA |  |  |
| Medium Metro | | 1.0 (-0.0, 1.9) | 1.0 (-0.0, 1.9) | 1999-2020 | NA | NA |  |  |
| Small Metro | | 1.6 (-0.0, 3.3) | 1.6 (-0.0, 3.3) | 1999-2020 | NA | NA |  |  |
| Micropolitan (Nonmetro) | | 0.4 (-0.7, 1.5) | 0.4 (-0.7, 1.5) | 1999-2020 | NA | NA |  |  |
| Noncore (Nonmetro) | | -0.2 (-1.9, 1.4) | -0.2 (-1.9, 1.4) | 1999-2020 | NA | NA |  |  |
|  | **statistically significant trend in TCSM | | | | | | | |

e**Table 5**. Testicular Cancer Mortality Place of Death in the US by Age, Marital Status, Educational Level, Race, and Ethnicity

|  | **Home** | **Hospice** | **Medical Facility** | **Total** |
| --- | --- | --- | --- | --- |
| **All** | **2317** | **466** | **4143** | **6926** |
| **Age group, y** |  |  |  |  |
| Birth-24 | 236 (27.63) | 34 (3.98) | 584 (68.38) | 854 |
| 25-44 | 1060 (31.5) | 188 (5.59) | 2117 (62.9) | 3365 |
| 45-64 | 640 (33.68) | 154 (8.11) | 1106 (58.2) | 1900 |
| 65-84 | 313 (46.4) | 75 (11.1) | 286 (42.4) | 674 |
| >85 | 68 (51.1) | 15 (11.3) | 50 (37.6) | 133 |
| **Marital Status** |  |  |  |  |
| Single | 836 (27.5) | 192 (6.3) | 2018 (66.3) | 3046 |
| Married | 1168 (40.0) | 192 (5.68) | 1557 (53.4) | 2917 |
| Widowed | 62 (37.4) | 21 (12.7) | 83 (50.0) | 166 |
| Divorced/separated | 251 (31.5) | 61 (7.65) | 485 (60.9) | 797 |
| **Educational level** |  |  |  |  |
| Some High School or less | 465 (31.0) | 85 (5.66) | 951 (63.4) | 1501 |
| High School Graduate (>4y) | 974 (33.2) | 203 (6.93) | 1754 (59.8) | 2931 |
| Some College/Associate’s Degree | 465 (34.7) | 107 (7.99) | 767 (57.3) | 1339 |
| College Graduate (>4y) | 351 (35.7) | 57 (5.80) | 575 (58.5) | 983 |
| Advanced Degree | 62 (36.1) | 14 (8.14) | 96 (55.8) | 172 |
| **Race** |  |  |  |  |
| White | 2159 (34.4) | 426 (6.79) | 3685 (58.8) | 6270 |
| Black or African American | 100 (23.9) | 26 (6.22) | 292 (69.9) | 418 |
| Asian or Pacific Islander | 43 (26.2) | 11 (6.71) | 110 (67.1) | 164 |
| American Indian or Alaska Native | 15 (20.3) | 3 (4.05) | 56 (75.7) | 74 |
| **Ethnicity** |  |  |  |  |
| Hispanic or Latino | 322 (24.5) | 81 (6.17) | 910 (69.3) | 1313 |
| Non-Hispanic | 1995 (35.5) | 385 (6.86) | 3233 (57.6) | 5613 |

|  | **eTable 6**. Annual Percentage Changes in Metastatic Testicular Cancer Incidence Rates by Age, Race, and Ethnicity from 1999 to 2018 | | | | | | | | |
| --- | --- | --- | --- | --- | --- | --- | --- | --- | --- |
| Demographic | | Average APC from 1999-2018 |  | Segment 1 |  | Segment 2 |  |  |  |
| All | | 1.0 (0.4,1.6)** | 1.0 (0.4,1.6)** | 1999-2018 | NA | NA |  |  |  |
| Age | |  |  |  |  |  |  |  |  |
| 20-24 | | 1.1 (0.5,1.7)** | 1.1 (0.5,1.7)** | 1999-2018 | NA | NA |  |  |  |
| 25-29 | | 1.6 (1.0,2.1)** | 1.6 (1.0,2.1)** | 1999-2018 | NA | NA |  |  |  |
| 30-34 | | 1.8 (1.2,2.4)** | 1.8 (1.2,2.4)** | 1999-2018 | NA | NA |  |  |  |
| 35-39 | | 0.6 (-0.1,1.3) | 0.6 (-0.1,1.3) | 1999-2018 | NA | NA |  |  |  |
| 40-44 | | 0.4 (-0.4,1.1) | 0.4 (-0.4,1.1) | 1999-2018 | NA | NA |  |  |  |
| 45-49 | | -0.6 (-1.8,0.6) | -0.6 (-1.8,0.6) | 1999-2018 | NA | NA |  |  |  |
| 50-54 | | 2.3 (1.0,3.6)** | 2.3 (1.0,3.6)** | 1999-2018 | NA | NA |  |  |  |
| **Race/Ethnicity** | |  |  |  |  |  |  |  |  |
| Hispanic | | 2.5 (2.0,3.1)** | 2.5 (2.0,3.1)** | 1999-2018 | NA | NA |  |  |  |
| Non-Hispanic White | | 0.6 (0.2,1.1)** | 0.6 (0.2,1.1)** | 1999-2018 | NA | NA |  |  |  |
| Non-Hispanic Black | | 0.4 (-1.2,2.0) | 0.4 (-1.2,2.0) | 1999-2018 | NA | NA |  |  |  |

**statistically significant trend in TCSI

|  | **eTable 7.** Annual Percentage Changes in Localized Testicular Cancer Incidence Rates by Age, Race, and Ethnicity from 1999 to 2018 | | | | | | | | |
| --- | --- | --- | --- | --- | --- | --- | --- | --- | --- |
| Demographic | | Average APC from 1999-2018 |  | Segment 1 |  | Segment 2 |  | Segment 3 |  |
| All | | -0.1 (-0.9, 0.6) | 0.2 (-0.1,0.5) | 1999-2011 | 1.7 (-0.1,3.6) | 2011-2016 | -6.6 (-11.8,-1.0)** | 2016-2018 |  |
| Age | |  |  |  |  |  |  |  |  |
| 20-24 | | 0.9 (0.5, 1.3)** | 0.9 (0.5,1.3)** | 1999-2018 | NA | NA | NA | NA |  |
| 25-29 | | 0.1 (-1.0, 1.2) | 1.4 (1.0,1.8)** | 1999-2016 | -10.0 (-19.2,0.2) | 2016-2018 | NA | NA |  |
| 30-34 | | 0.4 (0.0, 0.9)** | 0.4 (0.0,0.9)** | 1999-2018 | NA | NA | NA | NA |  |
| 35-39 | | -0.6 (-1.7, 0.6) | -1.2 (-1.9,-0.4)** | 1999-2009 | 2.1 (0.4,3.8)** | 2009-2016 | -6.4 (-15.2,3.3) | 2016-2018 |  |
| 40-44 | | -0.6 (-1.0, -0.2)** | -0.6 (-1.0,-0.2)** | 1999-2018 | NA | NA | NA | NA |  |
| 45-49 | | -0.4 (-0.7, 0.0) | -0.4 (-0.7,0.0) | 1999-2015 | NA | NA | NA | NA |  |
| 50-54 | | 0.2 (-0.5, 1.0) | 0.2 (-0.5,1.0) | 1999-2018 | NA | NA | NA | NA |  |
| 55-59 | | 1.8 (1.0, 2.7)** | 1.8 (1.0,2.7)** | 1999-2018 | NA | NA | NA | NA |  |
| 60-64 | | 1.8 (1.1, 2.6)** | 1.8 (1.1,2.6)** | 1999-2018 | NA | NA | NA | NA |  |
| 65-69 | | -0.4 (-1.4, 0.6) | -0.4 (-1.4,0.6) | 1999-2018 | NA | NA | NA | NA |  |
| 70-74 | | -0.3 (-1.5, 0.9) | -0.3 (-1.5,0.9) | 1999-2018 | NA | NA | NA | NA |  |
| 75-79 | | -1.9 (-3.2, -0.6)** | -1.9 (-3.2,-0.6)** | 1999-2018 | NA | NA | NA | NA |  |
| 80-84 | | -1.4 (-2.8, 0.0)** | -1.4 (-2.8,0.0)** | 1999-2018 | NA | NA | NA | NA |  |
| 85+ | | -2.0 (-3.7, -0.3)** | -2.0 (-3.7,-0.3)** | 1999-2018 | NA | NA | NA | NA |  |
| **Race/Ethnicity** | |  |  |  |  |  |  |  |  |
| Hispanic | | 2.2 (1.9, 2.6)** | 2.2 (1.9,2.6)** | 1999-2018 | NA | NA | NA | NA |  |
| Non-Hispanic White | | -0.1 (-0.9, 0.6) | 0.4 (0.1,0.7)** | 1999-2016 | -4.5 (-11.1,2.5) | 2016-2018 | NA | NA |  |
| Non-Hispanic Black | | 1.0 (0.4, 1.6)** | 1.0 (0.4,1.6)** | 1999-2018 |  |  |  |  |  |

 **statistically significant trend in TCSI

**eFigure 1. Age-Adjusted Rates of Testicular Cancer-Specific Mortality Over Time by Census Region**

Among all census regions, there was no significant shift in age-adjusted rate of TCSM over the study period from 1999 to 2020. There was an insignificant increase in age-adjusted rate of TCSM in the Midwest, South, and West census regions, while the Northeast experienced a slight decrease in TCSM (all P>0.05).

**eFigure 2. Age-Adjusted Rate of Testicular Cancer-Specific Mortality (TCSM) Over Time by Metropolitan Area**

Large fringe, medium metro, small metro, and micropolitan experienced a slight increase in TCSM rates (P>0.05). Large Central and NonCore were the only metropolitan areas to experience a slight decrease in TCSM rates (P>0.05).
